# ZapE/Afg1 interacts with Oxa1 and its depletion causes a multifaceted phenotype

**DOI:** 10.1371/journal.pone.0234918

**Published:** 2020-06-24

**Authors:** Jan Pyrih, Vendula Rašková, Ingrid Škodová-Sveráková, Tomáš Pánek, Julius Lukeš

**Affiliations:** 1 Institute of Parasitology, Biology Centre, Czech Academy of Sciences, České Budějovice (Budweis), Czech Republic; 2 Faculty of Science, University of South Bohemia, České Budějovice (Budweis), Czech Republic; 3 Faculty of Sciences, Comenius University, Bratislava, Slovakia; 4 Faculty of Sciences, University of Ostrava, Ostrava, Czech Republic; University of Cambridge, UNITED KINGDOM

## Abstract

ZapE/Afg1 is a component of the inner cell membrane of some eubacteria and the inner mitochondrial membrane of eukaryotes. This protein is involved in FtsZ-dependent division of eubacteria. In the yeast and human mitochondrion, ZapE/Afg1 likely interacts with Oxa1 and facilitates the degradation of mitochondrion-encoded subunits of respiratory complexes. Furthermore, the depletion of ZapE increases resistance to apoptosis, decreases oxidative stress tolerance, and impacts mitochondrial protein homeostasis. It remains unclear whether ZapE is a multifunctional protein, or whether some of the described effects are just secondary phenotypes. Here, we have analyzed the functions of ZapE in *Trypanosoma brucei*, a parasitic protist, and an important model organism. Using a newly developed proximity-dependent biotinylation approach (BioID2), we have identified the inner mitochondrial membrane insertase Oxa1 among three putative interacting partners of ZapE, which is present in two paralogs. RNAi-mediated depletion of both ZapE paralogs likely affected the function of respiratory complexes I and IV. Consistently, we show that the distribution of mitochondrial ZapE is restricted only to organisms with Oxa1, respiratory complexes, and a mitochondrial genome. We propose that the evolutionarily conserved interaction of ZapE with Oxa1, which is required for proper insertion of many inner mitochondrial membrane proteins, is behind the multifaceted phenotype caused by the ablation of ZapE.

## Introduction

Most eukaryotes retain a highly complex mitochondrion, which became an essential component of the cell by harboring the enzymatic machinery for ATP production and other important metabolic pathways [1]. Recent studies of mitochondria from non-model and neglected eukaryotic microorganisms revealed that their organelle contains several proteins or even complex metabolic pathways that are not present in the model eukaryotes. Mitochondrial FtsZ division machinery and the Type II secretion system may serve as such recently documented examples [2,3]. At the same time, many conserved proteins found in the mitochondria across a broad range of eukaryotes including animals, yeast, and/or plants still await their functional characterization.

ZapE (alternatively Afg1 in yeast or LACE1 in humans) is an ATPase located in the inner membrane of the human and yeast mitochondria, and in the inner membrane of *Escherichia coli* [4,5]. The depletion of the human ZapE homolog triggers morphological changes of the mitochondria, eventually leading to their fragmentation [6]. A somewhat similar phenotype was observed in bacteria, where the affected cells became elongated following the up- or down-regulation of ZapE [5]. The available data is compatible with the view that in bacteria, ZapE is part of the FtsZ division machinery [5]. However, the function of human and yeast homologs may be different, as it was proposed that their ZapE orthologs mediate degradation of the mitochondrially-encoded subunits of the respiratory complex IV [4,6]. Furthermore, both organisms lack the FtsZ division system [3]. ZapE was shown to mediate the translocation of p53 and subsequent apoptosis in humans [7]. It is also noteworthy that ZapE was highly affected in the proteomic survey of the Oxa1 depletome in humans [8]. Moreover, the functional link between Oxa1 and ZapE was also suggested in yeast, where only the mitochondrially-encoded subunits of respiratory complex IV were affected after the depletion of ZapE. Finally, a novel role for this protein in maintaining mitochondrial matrix proteostasis was suggested [9]. All in all, the phenotypes associated with ZapE in different organisms vary widely, and so far, they have not been integrated into a coherent picture.

*Trypanosoma brucei* is both an important human pathogen causing African sleeping sickness and a model organism with highly developed molecular biology tools. It contains a single reticulated mitochondrion with its own genome represented by a network of mutually catenated DNA circles, termed kinetoplast DNA (kDNA) [10]. Transcripts of several kDNA-encoded genes become translatable only after they undergo extensive RNA editing of the uridine insertion/deletion type [11]. Another unique feature of the *T*. *brucei* mitochondrion is its capacity to undergo massive morphological and structural changes in the course of the parasite’s life cycle, which involves vertebrate hosts and the tse-tse fly vector [12,13]. To shed light on the function(s) of the conserved ZapE protein, so far examined only in bacteria and opisthokonts, we have probed the function and interactions of its two paralogs, ZapE1 (XP_823041.1, Tb927.7.6930) and ZapE2 (XP_846313.1; Tb927.10.8070). According to the ATOM40 depletome-based mitoproteome [13] and Tryptag *in-situ* tagging database [14], both proteins are genuine components of the *T*. *brucei* mitochondrion.

A range of recently developed techniques that take advantage of proximity biotinylation, such as BioID, TurboID, and APEX, are particularly suitable for the studies of protein-protein interactions [15,16]. As compared to classical co-immunoprecipitation, their advantage is higher reproducibility and capacity to identify both stable complexes and transient interactions. Briefly, the protein of interest is fused with modified biotin ligase, which promiscuously biotinylates proteins in its proximity. However, until now BioID has been used in *T*. *brucei* only in just a handful of studies [17,18]. Here, we took advantage of the recently developed advanced biotin ligation-based approach named BioID2, which we have successfully adapted for *T*. *brucei*. In contrast to the classical BioID technique, BioID2 biotin ligase is smaller (26 kDa), more specific, and attached to the protein of interest by several nm-long linker, which improves protein folding and the biotinylation range [19]. When applied to *T*. *brucei*, BioID2 labeling produced a highly specific output. Consistent with human and yeast, Oxa1 was identified among three putative interaction partners of ZapE2. As *T*. *brucei* is very distantly related to opisthokonts, we hypothesize that interaction of ZapE and Oxa1 is conserved in eukaryotes. This view is further supported by the co-occurrence of ZapE and Oxa1 genes and by the α-proteobacterial origin of eukaryotic ZapE, as documented by extensive phylogenetic analysis.

## Results

### Proximity-dependent biotinylation in *T*. *brucei*

**Fig 1:**
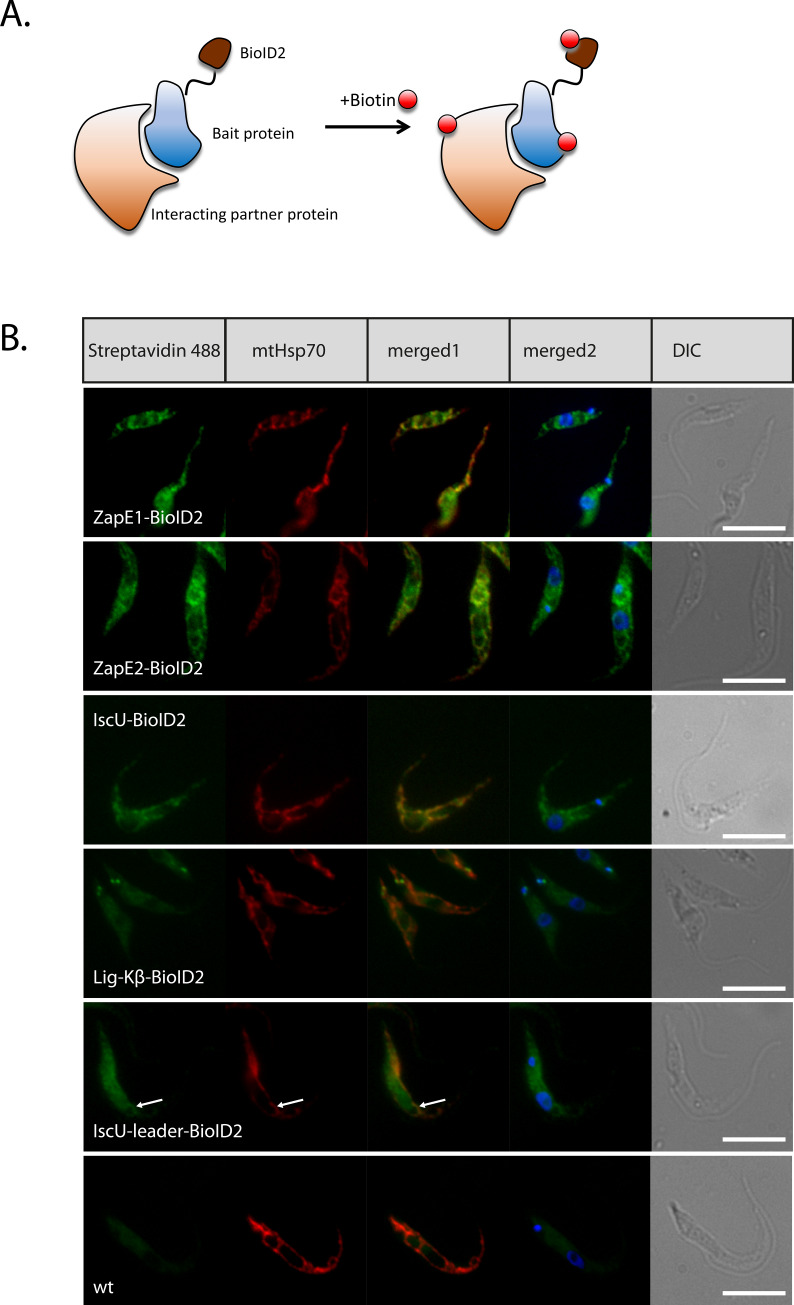
Mitochondrial proteins *in situ* tagged with BioID2. (**A**) A scheme of *in vivo* proximity-dependent biotinylation reaction induced by the addition of biotin. (**B**) Specific biotinylation for ZapE1, ZapE2, IscU, LigK-β, and IscU_Leader BioID2-HA fusion constructs. Fluorescently labeled Alexa-488 streptavidin detects biotinylated proteins in the mitochondrion (ZapE1, ZapE2, IscU), the antipodal kDNA sites (LigK-β) or predominantly in the cytoplasm (IscU_Leader BioID2 fusion). wt, wild type cells served as a control for background biotin staining. Monoclonal α-mHsp70 antibody was used as a mitochondrial marker. Arrow indicates the area in which partial mitochondrial localization of the IscU_Leader BioID2 fusion is visible. DNA was labeled with DAPI. DIC, differential interference contrast. Scale bars, 5 μm.

In order to establish the *in situ* proximity biotinylation approach in *T*. *brucei*, the mNeongreen gene was replaced with the HA-tagged modified biotin ligase from *Aquifex aeolicus* (BioID2) in the pPOTv7_mNG-Blast plasmid [14]. To increase the range of proximity biotinylation [19], we have designed ~8 nm-long glycine-serine (GGGGS) repeat-containing linker by which biotin ligase becomes linked with the protein of interest (Fig 1A). This pPOT_BioID2 plasmid served as a template for PCR-based C-terminal *in situ* tagging of the ZapE1 and ZapE2 genes in *T*. *brucei*. IscU, a chaperone involved in iron-sulfur (Fe-S) cluster assembly in *T*. *brucei* [20], and ligase K-β (LigK-β), which is critical for the kDNA replication and is located in the antipodal sites of the *T*. *brucei* kDNA network [21], were used as controls. Similarly, the mitochondrial import signal of the IscU gene was *in situ*-tagged with biotin ligase to serve as an additional control. For the *in situ* tagging strategy, pPOT_BioID2 plasmid map, and the full sequence of BioID2 protein with linker see S1 Fig.

The mitochondrial localization of the BioID2-tagged proteins and their ability to biotinylate surrounding proteins was confirmed by direct fluorescent Alexa-488-streptavidin labelling of the procyclic stage of *T*. *brucei* (Fig 1B). This streptavidin-fluorescent conjugate detected specific biotinylation in the mitochondrial lumen of the ZapE1, ZapE2, and IscU cell lines. In the case of LigK-β, the labelling occurred in the two opposing antipodal sites of the kDNA disk (Fig 1B). The leader sequence of IscU was insufficient for the delivery of the BioID2 protein into the organelle, resulting in a predominantly cytosolic localization (Fig 1B).

### Identification of mitochondrial proteins using BioID2

**Fig 2:**
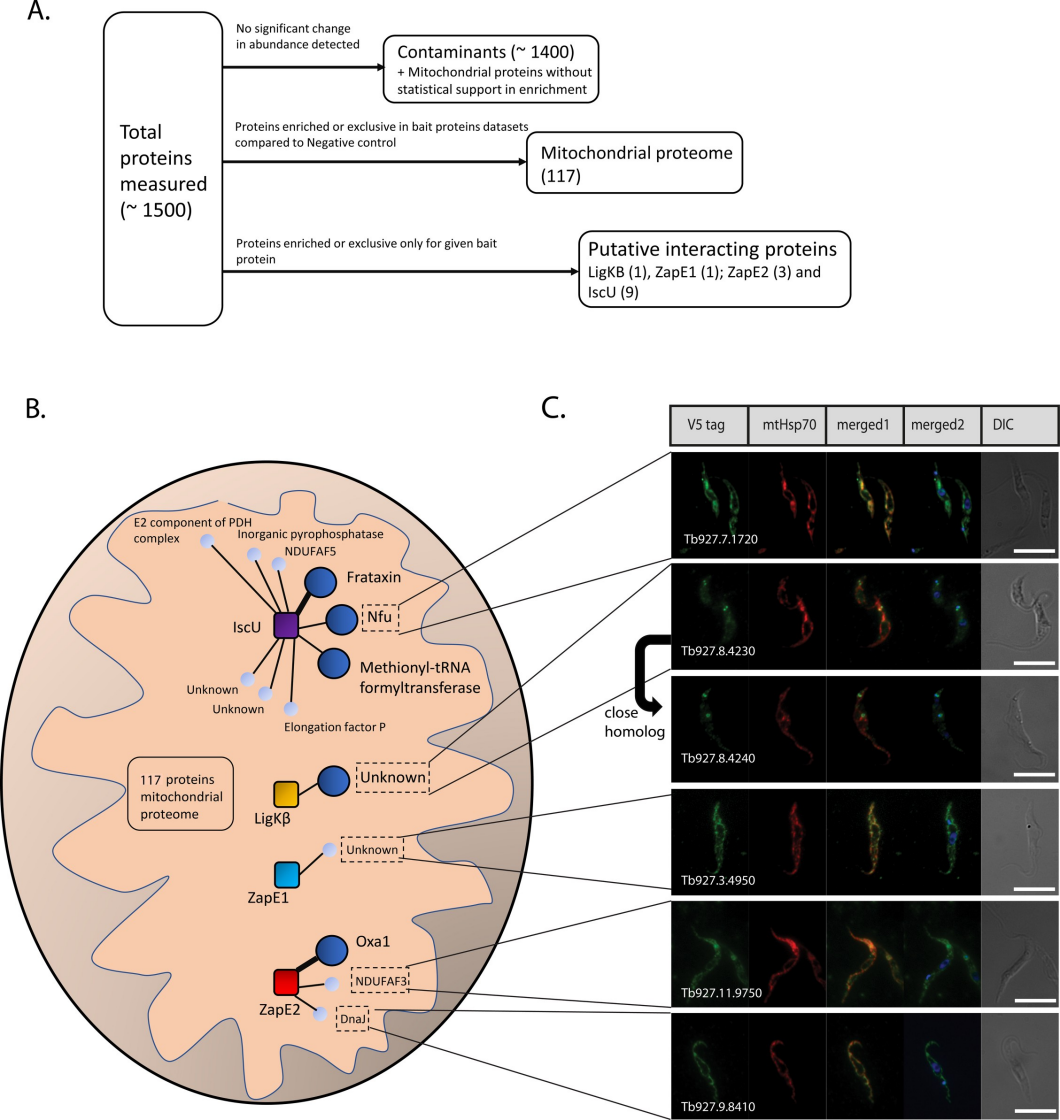
BioID2-labelled putative interacting proteins. (**A**) Scheme of how the proteomic data was processed to identify the mitochondrial proteome and putative interacting proteins. (**B**) Schematic representation displays identified putative interacting proteins. Each bait protein is represented by a square and is surrounded by identified putative interacting proteins visualized by a circle. The larger size of the circle for identified proteins highlights those exclusively identified only for a given bait protein or proteins enriched on average more than 100x as compared to other datasets. A thick line represents the expected interaction of the identified proteins based on data from other organisms. Proteins surrounded by the dashed rectangle are experimentally localized for the first time in this study. (**C**) Intracellular localization of proteins identified by the BioID2 technique. *In situ* C-terminally V5-tagged proteins were expressed in *T*. *brucei* and their localization was inspected using immunofluorescence microscopy. Monoclonal α-V5 rabbit and α-mHsp70 mouse antibodies were used. DNA was stained with DAPI. DIC, differential interference contrast. Scale bars, 5 μm.

An intrinsic feature of BioID is that very abundant proteins become biotinylated not only because of their specific interaction with the bait protein but also due to their by-chance proximity. Therefore, we focused on proteins enriched or exclusively present in at least three out of four analyzed BioID2 samples, namely ZapE1, ZapE2, IscU, and LigK-β when compared to the IscU-leader negative control (Fig 2A). The mitochondrial import signal of IscU-containing BioID2 construct was used as a negative control for proximity-dependent biotinylation, as most of the fusion protein remained in the cytoplasm (Fig 1B). To identify mitochondrial proteins, purified mitochondria of the *T*. *brucei* procyclic stage were dissolved in 1% SDS-containing buffer and incubated at 80°C for 10 min, after which solubilized biotinylated proteins were affinity-purified by streptavidin-coated Dynabeads. For each protein, three independent biological replicates have been performed. The protein composition of each affinity purification was determined by label-free quantitative proteomics.

A comparative analysis revealed a set of 117 mitochondrial proteins (S1 Table). The identified set primarily contains strongly expressed mitochondrial proteins such as mtHsp70 and Cpn60, which due to their proximity to the BioID2-tagged protein apparently became randomly biotinylated. In total, 114 out of 117 proteins have previously been listed in the mitochondrial proteome or shown as located within the organelle *via* the *in situ* tagging approach [13,14]. The remaining three proteins most likely also reside in the organelle, as they are either strongly predicted to be targeted into it or are homologs of known mitochondrial proteins (S1 Table). In the IscU-leader control, a small fraction was delivered into the organelle, yet in this negative control the detected mitochondrial proteins were on average 60 times less abundant as compared to bait proteins datasets (S1 Table).

### Identification of interacting proteins using BioID2

To distinguish between putative interacting partners and spurious interactions with abundant mitochondrial proteins, such as mtHsp70, other bait protein interactomes were used as controls. For instance, for LigK-β, purified IscU, ZapE1, and ZapE2 datasets were considered as negative controls. Proteins exclusively measured or more than 3x enriched as compared to the control datasets were considered as putative interacting partners (Fig 2A). By employing this strategy, however, we may filter out proteins that interact with two or more bait proteins. While this is unlikely for IscU and LigK-β, which have very different functions, the existence of a subset of overlapping interacting partners off ZapE1 and ZapE2 was plausible. For this reason, these two datasets were not treated reciprocally as a negative control but were compared to the IscU and LigK-β datasets only. As expected, the bait proteins invariably occupied the top position due to autobiotinylation by the fused BioID2 enzyme [19]. Along with the autobiotinylated protein, less than 10 significantly enriched proteins were identified for each analyzed bait (Fig 2B). For ZapE1 and LigK-β, only a single putative interacting protein was identified. A full list of proteins enriched both above and below the chosen threshold is available in S2 Table. Oxa1 was identified exclusively in the ZapE2 interactome. The other two proteins likely interacting with ZapE2 are homologs of NADH:ubiquinone oxidoreductase complex assembly factor 3 (NDUFAF3) and DnaJ chaperone, both *bona fide* components of the *T*. *brucei* respiratory chain complex I [22]. While NDUFAF3 was identified as a putative Oxa1-interacting protein in humans [23], the function of DnaJ chaperone in complex I of *T*. *brucei* is unknown, as homologs of this protein were so far not encountered associated with complex I in other eukaryotes.

IscU is a core component of the Fe-S cluster assembly pathway [11]. Three identified putative interacting partners–frataxin, *Tb*Nfu1, and Tb927.11.15470 (possible homolog of methionyl-tRNA formyltransferase) were exclusively associated with the IscU bait protein, with six additional proteins more than 3x enriched in the IscU dataset as compared to the other bait proteins. While frataxin is a well-known interacting partner of IscU in various organisms [24], a possible role of *Tb*Nfu1 in the early steps of the Fe-S cluster assembly pathway is intriguing. *T*. *brucei* contains three essential Nfu paralogs in its mitochondrion [25], which implies their unique specialization. While *Tb*Nfu2 and *Tb*Nfu3 likely function in the late steps of the Fe-S cluster pathway, as they were able to compensate for the depletion of their yeast homologs, the roles of *Tb*Nfu1 [25] and Tb927.11.15470 remain unknown. Finally, only one protein, Tb927.8.4230, putatively interacting with LigK-β was identified.

### Mitochondrial localization of putative interacting proteins

Most identified putative interacting proteins were either previously experimentally shown to localize to the mitochondrion of *T*. *brucei* [14] or are components of the mitochondrial ATOM40 depletome-based importome of the same organism [13]. Proteins for which intracellular distribution has not yet been convincingly shown were C-terminally *in situ*-tagged with the V5 tag and their mitochondrial localization was determined by immunofluorescence (Fig 2C). Thus we demonstrate that Tb927.11.9750 protein, a *bona fide* component of complex I [22], is beyond reasonable doubt localized within the organelle (Fig 2C). Moreover, Tb927.8.4230, a 119 kDa acidic protein (pI 5.4) is specifically confined to the antipodal sites of the kDNA disk, which is consistent with the localization of its interactor, LigK-β. Interestingly, an additional protein (Tb927.8.4240) identified by a BLAST search, seems to be a homolog of Tb927.8.4230 (e-value of 3e^-40^ in BLAST against *T*. *brucei* protein database). Genes for these homologs reside on the same chromosome next to each other. Surprisingly, Tb927.8.4240 is not localized directly in the antipodal sites of the kDNA disk, but in their proximity (Fig 1C).

### Depletion of ZapE proteins potentially affects the activity of complexes I and IV

**Fig 3:**
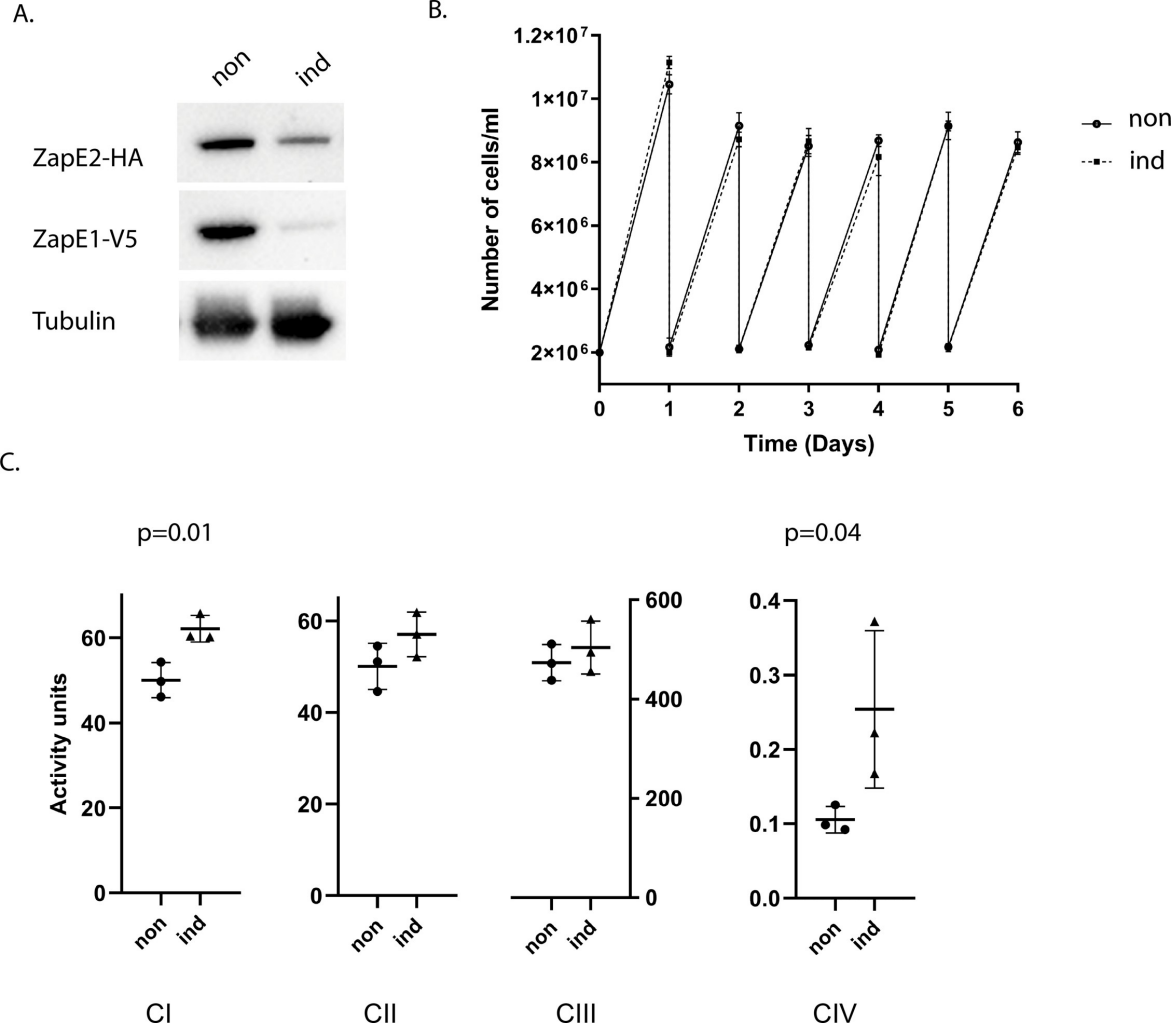
Downregulation of ZapE affects the activities of respiratory complexes. A double knock-down cell line was induced (ind) for six days with doxycycline. Noninduced (non) cells served as control. (**A**) ZapE1 and ZapE2 proteins were detected with α-V5 and α-HA antibodies, respectively, with α -tubulin antibody used as a loading control. (**B**) Cell densities of non-induced (circles) and RNAi-induced cells (squares) are indicated. The experiment was performed in biological triplicate. Error bars represent standard deviations. **(C)** Activities of two respiratory complexes are elevated in induced cells 6 days post-induction. The P-value of unpaired T-test is shown where a statistically significant difference was detected. Means from three independent biological replicates are displayed. Error bars represent standard deviation.

Initially, we created single RNAi knockdowns for either ZapE1 or ZapE2 in procyclic cells. While complete elimination of the respective V5-tagged targeted proteins (S2 Fig) was achieved in both cell lines, this was not associated with any measurable growth phenotype (S2 Fig). The most plausible explanation is that these conserved, yet individually non-essential proteins can functionally substitute each other. To test this hypothesis, we created a cell line in which both ZapE paralogs were downregulated by RNAi. To do that, ~500 nt-long regions of the ZapE1 and ZapE2 genes were cloned into the RNAi pTrypSon-Phleo and pTrypSon-Blast plasmids, respectively [26]. We then transfected both plasmids one at a time into a cell line in which ZapE1 and ZapE2 were *in situ*-tagged with the V5 and HA tags, respectively. The efficient ablation of the tagged proteins following RNAi induction was monitored by western blotting. While the signal for V5-tagged ZapE1 protein was virtually eliminated, RNAi was somewhat less efficient in the case of HA-tagged ZapE2 (Fig 3A). Unexpectedly, even tandem depletion of both ZapE paralogues had no effect on cell growth under standard cultivation conditions (Fig 3B).

Next, activities of two respiratory chain complexes were measured using spectrophotometric assays in the double knockdown (Fig 3C). Complex IV was affected most significantly, as its activity almost doubled in the ZapE proteins-depleted cells. A statistically significant upregulation of complex I activity was also observed, although to a lesser extent than that of complex IV. Putative changes in the activities of complexes II and III upon RNAi induction remained below statistical significance (Fig 3C). Prior to the set of measurements, the activities of respiratory complexes II, III, and IV were validated using their specific inhibitors, namely malonate, antimycin A, and KCN, respectively, which caused their near complete inhibition.

### Mitochondrial origin of ZapE in eukaryotes

**Fig 4:**
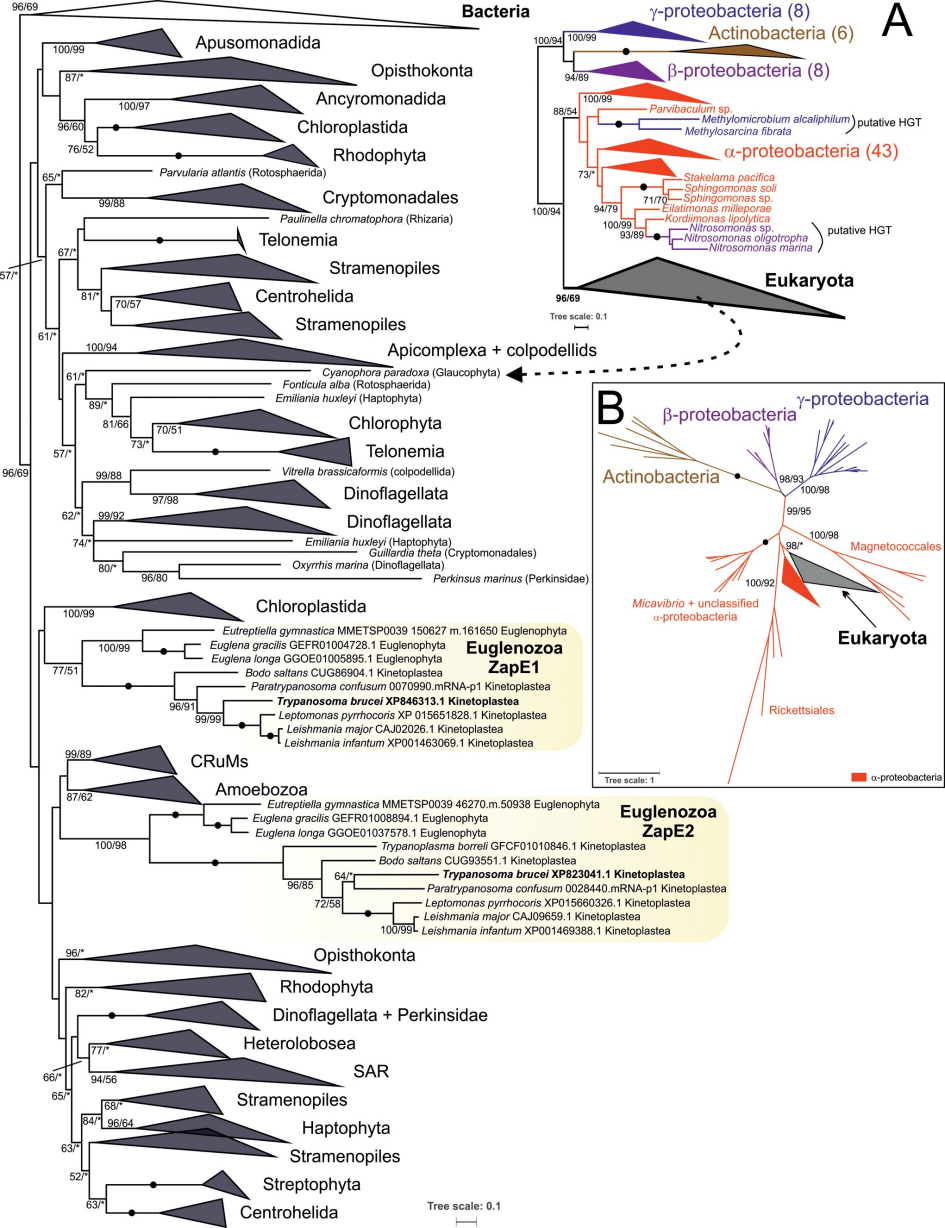
Phylogenetic analysis of ZapE. **(A)** A eukaryota-focused phylogenetic tree without long-branching α-proteobacterial sequences. The tree was arbitrary rooted using the midpoint-rooting method. Schematic representation showing the position of two proteobacterial clades branching within the clan of α-proteobacterial sequences is on the right, detailed phylogeny of eukaryotic ZapE on the left. A fully resolved tree is deposited as a supplementary S3 Fig. **(B)** Unrooted phylogenetic tree of ZapE that includes long-branching α-proteobacteria (Rickettsiales, Magnetococcales, *Micavibrio*) and shows a close affinity of eukaryotic ZapE to α-proteobacteria. Tree topologies are based on phylogenetic trees computed by the Maximum Likelihood method (LG4X model) in IQ-TREE. Branch supports were assessed by ultrafast bootstrap (N = 1000, IQ-TREE) and rapid bootstrap (N = 500, RAxML). Branch supports > 50% are indicated. A fully resolved tree is shown in S4 Fig.

To gain insight into the evolutionary origin of ZapE in eukaryotes, we carried out an extensive phylogenetic analysis, which includes eukaryotic as well as prokaryotic sequences (Fig 4). Outside eukaryotes, we identified ZapE only in two eubacterial groups, Actinobacteria and Proteobacteria. In our first analysis (Fig 4A), sequences formed four well-supported clades: i/ Actinobacteria (ultrafast bootstrap 100 and rapid bootstrap 100), ii/ β-proteobacteria (ultrafast bootstrap 94 and rapid bootstrap 89), iii/ γ-proteobacteria (ultrafast bootstrap 100 and rapid bootstrap 99), and iv/ α-proteobacterial and eukaryotic ZapE (support 100 and 94, respectively).

Eukaryotic sequences form a moderately supported group (ultrafast bootstrap 96, rapid bootstrap 69). The group of eukaryotic ZapE was formed in all preliminary analyses that differed in taxon composition and were independently trimmed. Moreover, the same topology was recovered by both IQ-Tree and RAxML, two phylogenetic programs for maximum likelihood analysis. Interestingly, no other ZapE genes were identified outside Actinobacteria, Proteobacteria, and Eukaryota. We also did not find any ZapE in other proteobacterial groups, namely δ- and ε-proteobacteria. Several ZapE sequences present in genomic assemblies of some prokaryotes were shown to be contaminants by reciprocal BLAST or by manual inspection of flanking genomic regions. In all cases, the most probable source of contamination was α-, β- or γ-proteobacteria (S3 Table). Several sequences from *Nitrosomonas* spp. (β-proteobacteria), *Methylomicrobium alcaliphilum* and *Methylsarcina fibrata* (both γ-proteobacteria) that fell within the α-proteobacterial clade most likely represent horizontal gene transfer events (Fig 4A).

To better understand the origin of eukaryotic ZapE, we performed another phylogenetic analysis which also included extremely derived sequences of deep-branching α-proteobacteria (Rickettsiales, Magnetococcales, etc.). This analysis indicates that eukaryotic ZapE originated from Proteobacteria, most likely from a group branching within α-proteobacteria (Fig 4B). We hypothesize that ZapE entered the eukaryotic cell with the proto-mitochondrion and was transferred to the eukaryotic nucleus from the mitochondrial genome before modern eukaryotes diverged from their last common ancestor. Internal phylogeny of eukaryotic ZapE is not well resolved, although we encountered several instances of independent gene duplications—in Euglenozoa, the SAR supergroup, and Chloroplastida.

### ZapE co-occurs with respiratory complexes, Oxa1 and mitochondrial genome in eukaryotes

**Fig 5:**
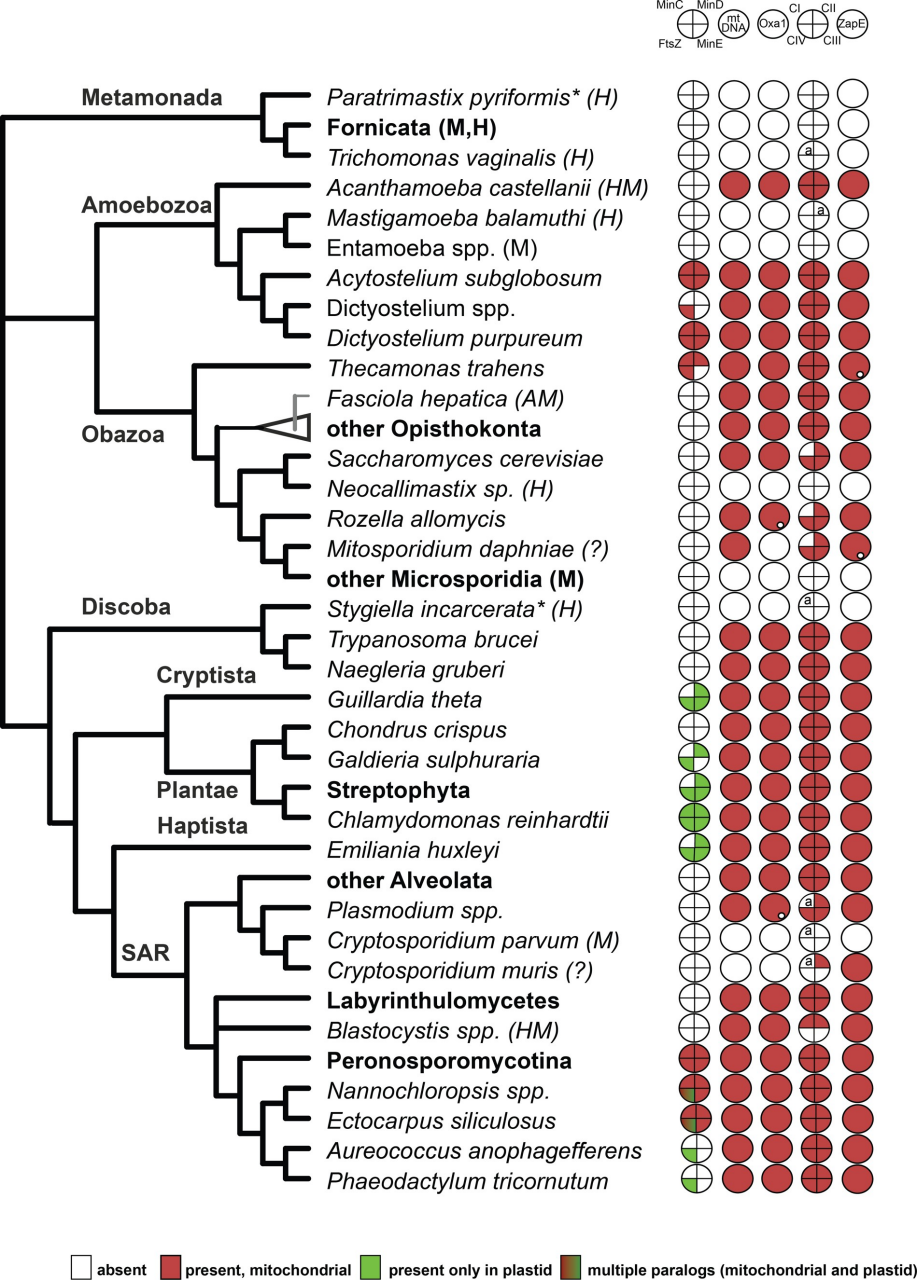
Co-occurrence of ZapE homologs with genes for respiratory complexes, Oxa1, and core FtsZ components. Prediction of mitochondrial/plastidial localization of given proteins based on Mitofates [28] and Deeploc [29] prediction tools is displayed. Small white dots indicate Oxa1 and ZapE proteins with unclear cellular localization. Data about subcellular localization and the presence of Min and FtsZ proteins were acquired from [3]. For the prediction of localization of respiratory complexes, phylogenetic affinity with previously localized proteins was considered. Alb3, plastid homolog of Oxa1, was excluded from the table. Asterisks (*) indicate results based on transcriptomic data; the letter “a” specifies that only some subunits of a given respiratory complex are present. AM, anaerobic mitochondrion; HM, hydrogen-producing mitochondrion; H, hydrogenosome; M, mitosome; (?), unclassified mitochondrion. Collapsed nodes representing higher taxonomic groups are in bold.

ZapE modulates Z-ring stability during the FtsZ-dependent cell division in the γ-proteobacterium *E*. *coli*. It is not essential during growth under laboratory conditions, but in the anaerobic conditions or at temperatures over 37°C, the importance of ZapE becomes evident [5]. Although FtsZ-dependent division is present in most of the major groups of Eubacteria as well as in some Archaea, plastids, and mitochondria [27], our analyses show that the taxonomic distribution of ZapE is different. It is present only in a few eubacterial lineages but nearly all eukaryotes. More interestingly, eukaryotes often encode ZapE even when mitochondrial FtsZ-dependent division has been completely lost (Fig 5). The ZapE gene is present in eukaryotes with classical aerobic mitochondria, anaerobically functioning mitochondria (e.g. *Fasciola hepatica* and *Euglena gracilis*) and hydrogen-producing mitochondria (e.g. *Blastocystis hominis* and *Acanthamoeba castellanii*). On the other hand, it is absent in the obligate anaerobes equipped with extremely reduced derivates of mitochondria, such as hydrogenosomes and mitosomes (e.g. *Entamoeba histolytica* and *Giardia intestinalis*).

The presence of ZapE in eukaryotes depends on respiratory chain complexes, especially complex II, and correlates with the retention of the mitochondrial genome and Oxa1. *Cryptosporidium muris*, but not its close relative *C*. *parvum*, is the sole identified exception, as it retains the ZapE gene and respiratory complex II, while Oxa1 and most probably also the mitochondrial genome are absent [30] (Fig 5).

## Discussion

For ZapE, a spectrum of seemingly unrelated functions ranging from apoptosis to cell division has been proposed [5–7,9]. Curiously, it was not yet addressed how a single protein may perform such a plethora of functions. Here we propose a hypothesis, which connects most of the observed phenotypes into a single putative role of the ZapE protein.

One of the three identified putative interaction partners of the ZapE2 paralog in *T*. *brucei* is Oxa1, a highly conserved protein required for both the insertion of mitochondrially-encoded subunits of the respiratory chain complexes [31], as well as of 28 nuclear-encoded proteins residing in the inner mitochondrial membrane, such as mitochondrial carriers, Atm1, and the sdh3 and sdh4 subunits of complex II [8]. We suggest that in eukaryotes Oxa1 and ZapE operate together, and that a depletion of the latter partner triggers a complex phenotype.

In yeast, only the mitochondrially-encoded subunits (cox1, cox2, and cox3) of complex IV were affected by the ablation of ZapE [4]. This observation can be nicely explained by a functional linkage between ZapE and Oxa1. Furthermore, ZapE was recently identified among the substrates of Oxa1, as its expression was dramatically decreased in the Oxa1 knock-down cells [8]. However, among 28 proteins identified in this survey, ZapE was the only one lacking a transmembrane domain, strongly indicating that it is not an integral protein of the inner mitochondrial membrane. This implies that ZapE is not a substrate of Oxa1 but rather its interaction partner. Finally, ZapE seems to be invariably present in the aerobic mitochondria (Fig 5), a key feature of which is the presence of numerous subunits of the respiratory chain complexes encoded by the mitochondrial genome [32]. Their protein products are inserted with the assistance of the membrane insertase Oxa1 into the corresponding complexes residing in the inner membrane [33]. In contrast, we have noted that ZapE is prominently absent in anaerobes lacking the mitochondrial genome, respiratory complexes, and Oxa1.

In *T*. *brucei* in which both ZapE paralogs were downregulated, the activities of Oxa1 substrates, in particular complexes I and IV, significantly increased. In yeast it was shown that following the depletion of ZapE, mitochondrially-encoded complex IV subunits cox1, cox2, and cox3 were also slightly elevated, which led the authors to conclude that ZapE facilitates their degradation [4]. As Oxa1 participates in the insertion of several subunits of the respiratory complexes into the inner mitochondrial membrane, we propose that ZapE may negatively regulate the function of Oxa1. We attribute the lack of growth phenotype to the fact that a small fraction of the target proteins escaped RNAi, but alternatively it is possible, that proteins might not be required for normal cell growth under tested conditions. Nevertheless, our findings are consistent with the connection between ZapE/Afg1 and the respiratory chain, which was so far observed only in yeast and humans [4,6].

We then investigated how the interaction of ZapE with Oxa1 could be linked with other described roles of the former protein, which was associated with the FtsZ-dependent bacterial division, as both its overexpression and ablation resulted in elongated bacterial cells [5]. The FtsZ-dependent division system, which comprises at least four components, was retained in the inner membranes of mitochondria and plastids of various eukaryotes, such as *Dictyostelium purpureum*, *Malawimonas californiana*, *Guillardia theta*, *Chondrus crispus* and *Chlorella variabilis* [3]. We explored a possible co-occurrence of ZapE with the FtsZ machinery, but none was found, so it is likely that ZapE participates in different processes. In phylogenetic trees, eukaryotic ZapE homologs form a sister group to α-proteobacterial ZapE, implying their mitochondrial origin, a conclusion further supported by the absence of ZapE in plastids and cyanobacteria (Fig 4). Therefore, the function of ZapE was either completely changed at an early stage of the eukaryotic evolution or, alternatively, YidC (a homolog of mitochondrial Oxa1) is an interaction partner of ZapE also in bacteria. Another conclusion based on the phylogenetic analysis is that the two ZapE paralogs appeared before the radiation of kinetoplastid flagellates. Since the only identified putative interactor of ZapE1 is a protein of unknown function (Tb927.3.4950) with no homology outside the kinetoplastids, it does not provide any information about the function of ZapE1, which will possibly be specific for this group of highly derived protists. However, we cannot rule out a scenario, in which Oxa1 interacts with both paralogs, yet the interaction with ZapE1 is more transient or was disrupted by tagging.

In mammalian cells, the depletion of ZapE resulted in fragmented mitochondria [6], a phenotype reminiscent of that seen during deficiency in respiratory complexes [34]. A key component of the mitochondrial cristae shaping complex (MICOS) [35], Mic10 belongs to the substrates of Oxa1 [8]. Therefore, it is possible that the observed impact of ZapE on mitochondrial and bacterial morphology is just a consequence of the destabilization of respiratory chain components and/or the MICOS complex. Similarly, we can easily link ZapE and Oxa1 with apoptosis, as the respiratory chain complexes are its modulators [36], as well as with proteostasis since Oxa1 is responsible for the insertion of a subset of the mitochondrially- and nuclear-encoded inner membrane proteins [8].

To further validate the use of the BioID2 *in vivo* labelling technique, we turned our attention to IscU and LigK-β, which were used as controls. Frataxin and TbNfu1 (both components of the Fe-S cluster pathway) were among the three proteins exclusively associated with IscU. Quite surprising, however, is the absence of IscS, which was proposed to interact with IscU in trypanosomes [37]. This protein was strongly but equally measured in all bait protein datasets. It is possible that the fusion of IscU with BioID2 disrupted its structure and thus prevented its specific interaction with IscS.

For LigK-β, only Tb927.8.4230 was identified as a putative interacting protein, which was more than 100x enriched compared to other datasets. Importantly, this unknown function protein localized into the antipodal sites of the kDNA disk. Such a highly specific localization is telling. Due to its absence in the large non-catenated pro-kinetoplast DNA of the free-living *Bodo saltans* [38], it is plausible that this protein is involved in the catenation of circular DNAs, so-called minicircles, into the single kDNA network of *T*. *brucei*. Moreover, by a BLAST search, we found a 184 kDa homolog of Tb927.8.4230 named Tb927.8.4240. Reassuringly, the latter is also present in the proximity of the kDNA disk of trypanosomes, yet most likely does not reside in the antipodal sites (Fig 2B).

Additionally, we tested the suitability of BioID2 for the mapping of the mitochondrial proteins. We identified 117 proteins, all located to the organelle with high confidence. Thus, the BioID2 technique produces a very clean, albeit incomplete *T*. *brucei* mitochondrial proteome, which is estimated to contain ~1,200 proteins [13,14].

In conclusion, we have successfully established the BioID2 technique in *T*. *brucei*. The specificity of this technique was proven by a high purity of the mitochondrial protein dataset. Using this technique, we were able to demonstrate that Oxa1 is a putative interaction partner of the ZapE homolog also outside the Opisthokonta, which allowed us for the first time to identify common features of the ZapE-related phenotypes in two eukaryotic supergroups. More research is, however, needed to confirm the nature of the interaction between Oxa1 and ZapE proteins.

## Experimental procedures

### Preparation of cell lines

*T*. *brucei* procyclic stage SmOx cell line [39] was grown in SDM79 supplemented with 10% fetal bovine serum for most of the experiments. Alternatively, the glucose-poor SDM80 medium [40] was used for studies of the phenotype triggered by ZapE RNAi-based depletion. Various proteins were *in situ*-tagged by a recently developed PCR-based transfection protocol described elsewhere [14]. As a PCR template, different versions of the pPOT plasmids were used (S4 Table). For instance, proteins identified by the proteomic analysis and ZapE homologs were C-terminally V5-tagged using a previously modified pPOTv4_Hyg vector [41]. In order to *in situ* fuse various proteins with BioID2, pPOT7-Blast-mNG plasmid (kindly provided by Samuel Dean) was modified by replacing the mNG gene with the BioID2 gene so that the protein of interest is linked with the BioID2 protein by an 8 nm-long glycine-serine repeats-containing linker (S1 Fig). Furthermore, long hairpin RNAi constructs were assembled for the ZapE1 and ZapE2 double RNAi knock-down by cloning two 450 nt-long regions into the pTrypSon plasmid by the Gibson assembly protocol as described previously [26]. Note that the pTrypSon plasmid with long hairpin ZapE2 was further modified by exchanging the phleomycin resistance with that of blasticidin. In the double RNAi knock-down cells, ZapE1 and ZapE2 were *in situ*-tagged with V5 tag and HA tag, respectively, and then both proteins were targeted using the dedicated pTrypSon plasmids. This strategy resulted in a double knockdown cell line modified by four plasmids with four different resistances (DKD cell line). A similar strategy was employed to create single knockdown cell lines (S4 Table).

### RNAi phenotype analysis

Cultures were grown in triplicate in the presence or absence of doxycycline. Cell density was counted using the Beckman Coulter Z2 Cell and Particle Counter every 24 hours and cells were subsequently diluted to 2 × 10^6^ cells/ml, maintaining them in the exponential phase of growth. For Western blot analysis, monoclonal α-V5, α-HA (Life Technologies), α-tubulin antibodies (Sigma-Aldrich), and secondary HRP-conjugated α-mouse and α-rabbit IgG antibodies (Sigma-Aldrich) were used and the signal was visualized by Clarity Western ECL Blotting Substrate (Bio-Rad).

### Enzymatic assays

For enzymatic assays, hypotonically isolated mitochondria from 5 × 10^8^ cells were lysed on ice for 1 hour in 2% (w/v) dodecyl maltoside and 0.4 M aminocaproic acid. Upon centrifugation at 24.400 g for 30 min at 4°C supernatant was used for activity measurements. All measurements were carried in biological triplicates.

Activities of respiratory enzymes (complexes I–IV) were measured by a spectrophotometric approach described previously [42]. Briefly, NADH dehydrogenase activity (complex I) was measured in 1 ml of NDH buffer (50 mM KPi, pH 7.5; 1 mM EDTA, pH 8.5; 0.2 mM KCN) with the addition of 0.1 mM NADH. The reaction was started by the addition of 2 μM coenzyme Q_2_ and followed at 340 nm for 3 min. For succinate dehydrogenase activity (complex II) mitochondrial lysate was pre-incubated with SDH buffer (25 mM KPi, pH 7.2; 5 mM MgCl_2_; 20 mM sodium succinate) at 25°C for 10 min. Next, antimycin A, rotenone, KCN, and 2,6-dichlorophenolindophenol (DPIP) were separately added to a final concentration of 1.8 mM, 5 mM, 2 mM, and 50 μM, respectively. The reaction itself was started by the addition of coenzyme Q_2_ to a final concentration of 65 μM and monitored at 600 nm for 5 min.

Cytochrome *c* reductase activity (complex III) was measured in 1 ml of QCR buffer (40 mM NaPi, pH 7.4; 0.5 mM EDTA; 20 mM sodium malonate; 50 μM cytochrome c; 0.005% [w/v] dodecyl maltoside). Simultaneously, 2 μl of mitochondrial lysate and 20 μM 2,3-dimethoxy-5-methyl-6-dodecyl-1,4-benzoquinol (DBH) were added. DBH was prepared by reduction of decylubiquinone as described elsewhere [43]. Reaction was followed at 550 nm for 1 min. Cytochrome *c* oxidase activity was measured in 1 ml of COX buffer (40 mM NaPi, pH 7.4; 0.5 mM EDTA; 20 μM cytochrome c; 30 μM ascorbic acid; 0.005% [w/v] dodecyl maltoside; solution was incubated overnight to oxidize surplus of ascorbic acid).

The mitochondrial lysate was added to the reaction buffer and monitored at 550 nm for 10 min. The unit (U) of appropriate activity is defined as an amount of enzyme required for the conversion of 1 nmol of NADH/min for NADH dehydrogenase; 1 nmol of DPIP/min for succinate dehydrogenase; 1 μmol of cytochrome *c* for both cytochrome *c* reductase and cytochrome *c* oxidase activities. Specific activities for all measurements were calculated as U per mg of mitochondrial proteins. To confirm the specificity of each enzymatic measurement, inhibitors of respiratory complexes were used. 1 mM sodium malonate, antimycin A at final concentration 300 ng/ml, and 100 μM KCN selectively inhibited the activities of complexes II, III, and IV, respectively. Inhibition of complex I was not tested since its contribution to overall NADH dehydrogenase activity within the cell is only partial [44].

### Immunofluorescence

Cells were fixed with 4% paraformaldehyde in phosphate buffered saline (PBS) and then permeabilized with 0.1% Triton X-100 in PBS. After blocking (1% BSA; 0.03% Triton X-100 in PBS) monoclonal rabbit α-V5 (Sigma-Aldrich), and monoclonal mouse α-mtHsp70 (kindly provided by Alena Zíková) was used followed by α-rabbit Alexa Fluor 488 and α-mouse Alexa Fluor 555 staining (Life Technologies). DNA was stained with ProLong^TM^Gold antifade reagent with 4',6-diamidine-2'-phenylindole dihydrochloride (DAPI) (Molecular Probes). The immunofluorescence assay was performed using a Zeiss microscope Axioplan 2 equipped with an Olympus DP73 digital camera.

### Proteomic identification by BioID2

Prior to the experiment, SDM79 media was supplemented overnight with 100 μM biotin. Then mitochondrion-enriched fraction was obtained from 3 × 10^9^ *T*. *brucei* procyclic cells by hypotonic lysis as described previously [42]. Mitochondria-enriched pellets were then resuspended in 1.8 ml of Boiling Buffer (1% SDS; 1 mM EDTA; 50 mM Tris; pH 7.4) for 10 min at 80°C. The dissolved samples were subsequently 10 times diluted in Incubation Buffer (150 mM NaCl; 5 mM EDTA; 1% Triton X-100; 50 mM Tris; pH 7.4) supplemented with Complete protease EDTA-free inhibitors (Roche). 0.5 mg of Dynabeads was added per sample (MyOne™ Streptavidin C1) and placed on a rocking platform for 2 hours at room temperature, following which the samples were stored overnight at 4°C. The Dynabeads were then washed three times separately with 1.5 ml of Boiling Buffer and 1.5 ml of Incubation Buffer. Dry Dynabeads were stored at -80°C prior to the proteomic analysis. The detailed protocol was deposited on protocol.io website (http://dx.doi.org/10.17504/protocols.io.bdrri556).

### Mass spectrometry

Proteins immobilized on Dynabeads were trypsin-digested and nanoflow liquid chromatography was used for separation of resulting peptides. Next, analysis of samples by tandem mass spectrometry (nLC-MS2) on a Thermo Orbitrap Fusion (q-OT-qIT, Thermo Scientific) instrument was performed as described elsewhere [45]. The mass spectrometry data were analyzed and quantified using MaxQuant software (version 1.5.3.8) [46] with false discovery rate set to 1% for both proteins and peptides and we specified a minimum length of seven amino acids. The Andromeda search engine was used for the MS/MS spectra search against the *Trypanosoma brucei* (downloaded from Uniprot, November 2018). Enzyme specificity was set as C-terminal to arginine and lysine, also allowing cleavage at proline bonds and a maximum of two missed cleavages. Dithiomethylation of cysteine was selected as fixed modification and N-terminal protein acetylation and methionine oxidation as variable modifications. The “match between runs” feature of MaxQuant was used to transfer identifications to other LC-MS/MS runs based on their masses and retention time (maximum deviation 0.7 min) and this was also used in quantification experiments. Then normalized intensity values were further processed using the Perseus software 1.5.2.4 [47]. Only proteins identified exclusively in each bait protein dataset or statistically significantly enriched were considered as putative interacting proteins. Exclusive identification is here defined as a situation where a given protein was measured in all three replicates of bait protein but was absent in all three control replicates. Statistically significant enrichment applies here for cases where a given protein was identified in all replicates for both, bait protein, and control, yet the difference between these groups was statistically significant. For statistical analysis, a Two-sample test with S0 parameter set to 0.1 was used as described elsewhere [47]. Mass spectrometry data have been deposited in the ProteomeXchange Consortium via the PRIDE [48] partner repository with the dataset identifier PXD014426.

### Bioinformatic analyses

To identify ZapE homologs in both eukaryotes and prokaryotes, we prepared an initial dataset via the BLASTp algorithm with human and *E*. *coli* ZapE sequences as queries against NCBI non-redundant protein "nr" database (https://www.ncbi.nlm.nih.gov/). Fifty best BLASTp hits for each query were kept, merged to a single dataset, deduplicated, and aligned by MAFFT version 7 (ginsi algorithm). The alignment was subsequently used to build Hidden Markov Model for a sensitive homolog search by HMMER3 software [49] to get candidate ZapE proteins from 100 strategically sampled eukaryotic genomes and transcriptomes. In addition, we also searched taxonomically restricted NCBI non-redundant protein databases for Archaea and Eubacteria, the latter clustered at 70% threshold (https://toolkit.tuebingen.mpg.de; psi-blast). Presence in deep-branching α-proteobacteria was specifically tested by search in available metagenomic and genomic data. All candidate protein sequences were then aligned by MAFFT version 7 in auto mode [50] and trimmed manually. Only well-aligned complete or nearly complete sequences were retained for further analysis. To remove identical sequences and to decrease the number of sequences in overrepresented lineages, preliminary phylogenetic analysis was performed in IQ-TREE multicore version 1.6.10 [51] with 1000 ultrafast bootstraps under the LG4X substitution model suggested by ModelFinder [52]. Subsequently, several rounds of reciprocal BLASTp and phylogenetic analyses were performed to remove contaminants (S3 Table). The two versions of the final protein dataset (Fig 4A and 4B) were aligned by MAFFT version 7, G-INS-i method with BLOSUM30 scoring matrix, and unaligned level 0.3 or 0.6, respectively. Both were visually inspected and trimmed manually. Maximum-Likelihood phylogenetic analysis was inferred with IQ-TREE as described above. Branch supports were estimated by rapid bootstrapping in RAxML version 8.12.11 [53] (LG4X model, 500 replicates) and ultrafast bootstrapping in IQ-TREE with activated “bnni” option to reduce the risk of overestimating branch supports (1,000 replicates). Bootstrap replicates were mapped on the best IQ-TREE topology and visualized by CorelDRAW Home & Student Suite X8.

## Supporting information

S1 Fig Scheme of the pPOT7_BioID2 plasmid, *in situ* tagging strategy, and the full sequence of BioID2 protein with the linker. The pPOT7_BioID2 plasmid served as a template for long-primer PCR. These primers have 20 bp homology to the plasmid at its 3´ end and 80 bp homology to the *T*. *brucei* genomic sequence at its 5´ end. This 80 bp homology regions facilitate specific recombination. The whole PCR product was then inserted in frame before the stop codon of the gene. The sequence of the BioID2 tag with GS linker, which replaced the stop codon of the gene, is also shown. PFR 3’, Paraflagellar rod protein 2 terminator; ald 5’ and 3’, aldolase promotor and terminator; bsr, blasticidin S deaminase gene.
(TIF)

S2 Fig Depletion of ZapE1 and ZapE2 protein expression and growth phenotype in single knockdown procyclic cell lines. Single knockdown ZapE1 and ZapE2 RNAi cells were treated with doxycycline for six days. (**A, C**) Protein levels detected by Western blot analysis. Non, without doxycycline; Ind, with doxycycline. α-tubulin antibody serves as a loading control. (**B, D**) Growth rates of induced and uninduced cell lines. The experiment was performed in biological triplicate. Error bars represent standard deviations.
(TIF)

S3 Fig Fully resolved rooted Eukaryota-focused phylogenetic tree of ZapE without long-branching α-proteobacterial sequences. (TIF)

S4 Fig Fully resolved unrooted phylogenetic tree of ZapE that includes long-branching α-proteobacteria. (PDF)

S1 Table Mitochondrial proteins of *T*. *brucei* identified by BioID2 approach. Column E shows the probability that a given protein has the mitochondrial import signal detected with the Mitofates online prediction tool. Columns F and G display whether a given protein was previously experimentally localized in the mitochondrion (TrypTag) or was present in the Tom40-based depletome. Columns J to Q show enrichment of a given protein in the bait protein datasets compare to IscU_leader_BioID2 negative control dataset. +, statistical significance (Two-sample test) of enrichment in the previous column. Columns R to AF display a Log_2_-transformed intensities for a given protein in a specific dataset. Lower case letters a, b, and c represent each replicate. Three proteins highlighted in blue were not present in the Tom40-based depletome, nor were they experimentally localized.
(XLSX)

S2 Table Full list of proteins significantly enriched by BioID2 labelling. Columns B and C: predicted function and e-value based on BLASTp algorithm against the NCBI non-redundant protein "nr" database (https://www.ncbi.nlm.nih.gov/against) with a parameter to exclude kinetoplastids. Columns D to G: statistically significant enrichment of a given protein against other bait protein datasets. “-”demarks when enrichment could not be calculated (*e*.*g*. when a given protein was measured only for a bait protein). Column M: the probability that a protein has the mitochondrial import signal detected with the Mitofates online prediction tool. Columns N and O display whether the protein was previously experimentally localized in the mitochondrion (TrypTag) or was present in the Tom40-based depletome. Columns P to AD display logarithm of two of measured intensities for a given protein in a specific dataset. Lower case letters a, b, and c represent each replicate. Proteins enriched on average less than three times are highlighted in blue.
(XLSX)

S3 Table Complete list of contaminant sequences, which were filtered out from the phylogenetic analysis. Reverse BLAST results for each gene are displayed in column D.
(XLSX)

S4 Table Used plasmids and cell lines. (XLSX)

S1 Raw Images (PDF)
