# Supplementary figures and images for: ZapE/Afg1 interacts with Oxa1 and its depletion causes a multifaceted phenotype
Source: PLoS One. 2020 Jun 24;15(6):e0234918. doi: 10.1371/journal.pone.0234918 (PMC7314023; doi:10.1371/journal.pone.0234918)

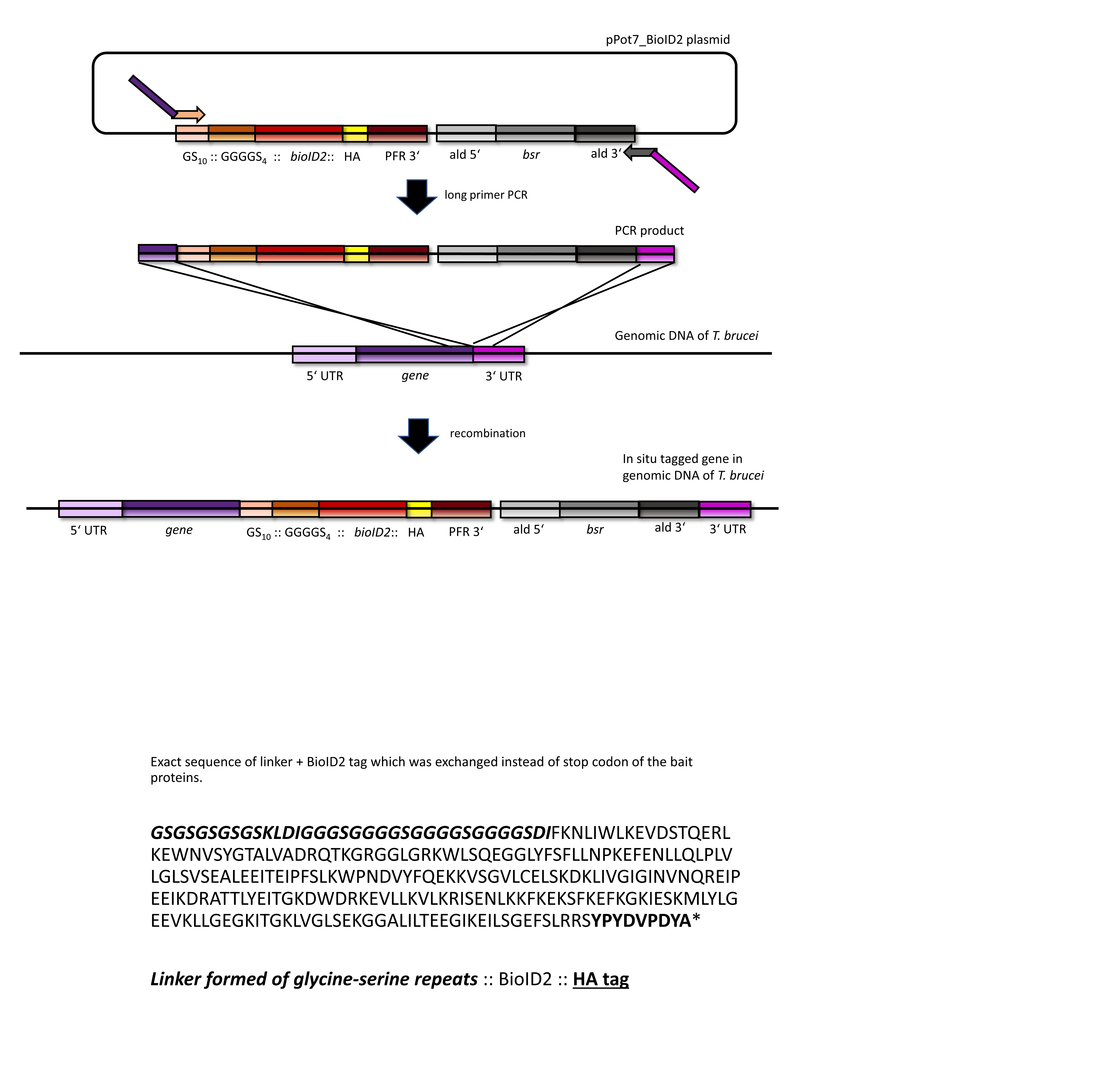

Supplement: S1 Fig — The pPOT7_BioID2 plasmid served as a template for long-primer PCR. These primers have 20 bp homology to the plasmid at its 3´ end and 80 bp homology to the T. brucei genomic sequence at its 5´ end. This 80 bp homology regions facilitate specific recombination. The whole PCR product was then inserted in frame before the stop codon of the gene. The sequence of the BioID2 tag with GS linker, which replaced the stop codon of the gene, is also shown. PFR 3’, Paraflagellar rod protein 2 terminator; ald 5’ and 3’, aldolase promotor and terminator; bsr, blasticidin S deaminase gene. (TIF) [file pone.0234918.s001.tif]

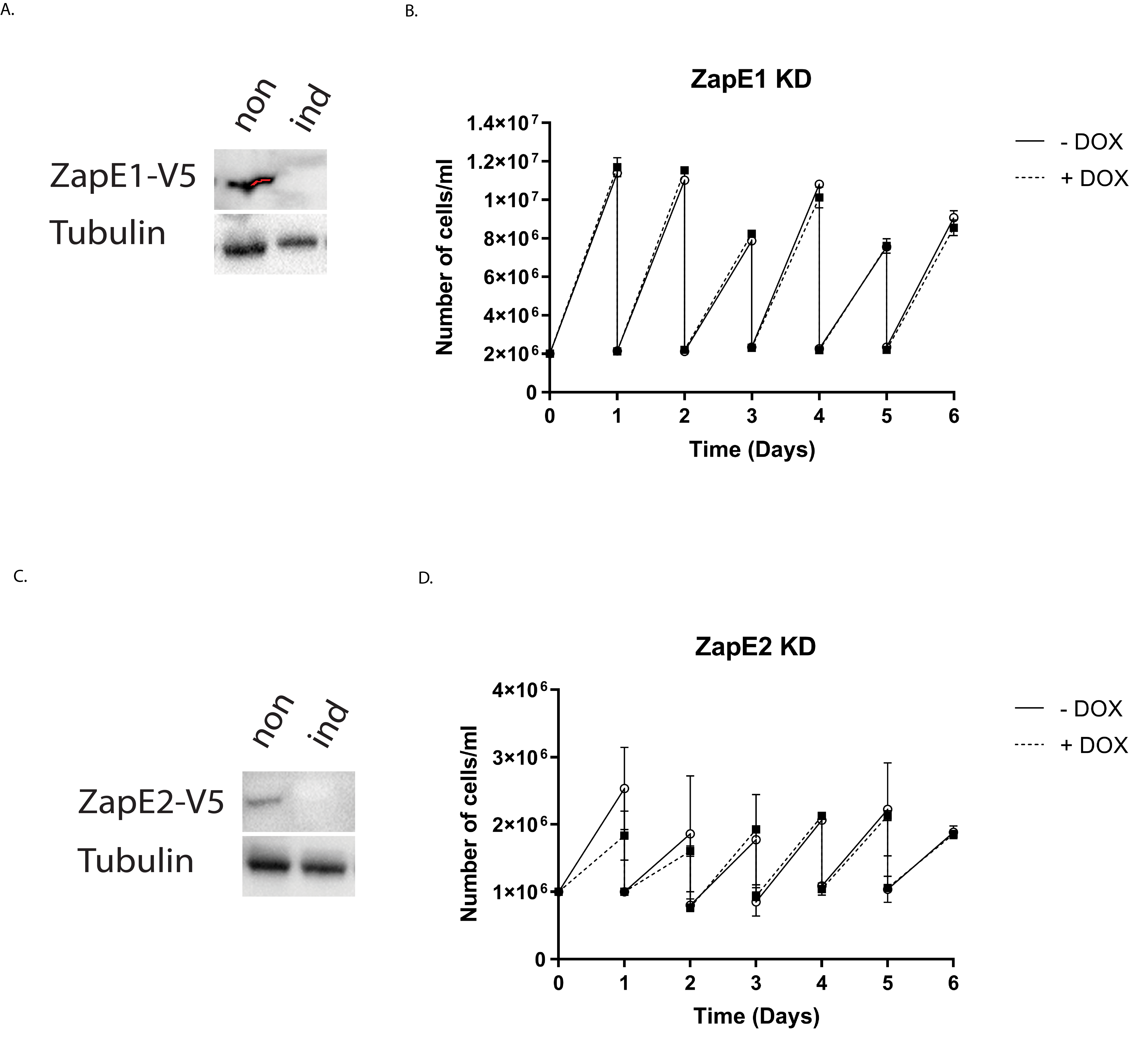

Supplement: S2 Fig — Single knockdown ZapE1 and ZapE2 RNAi cells were treated with doxycycline for six days. (A, C) Protein levels detected by Western blot analysis. Non, without doxycycline; Ind, with doxycycline. α-tubulin antibody serves as a loading control. (B, D) Growth rates of induced and uninduced cell lines. The experiment was performed in biological triplicate. Error bars represent standard deviations. (TIF) [file pone.0234918.s002.tif]

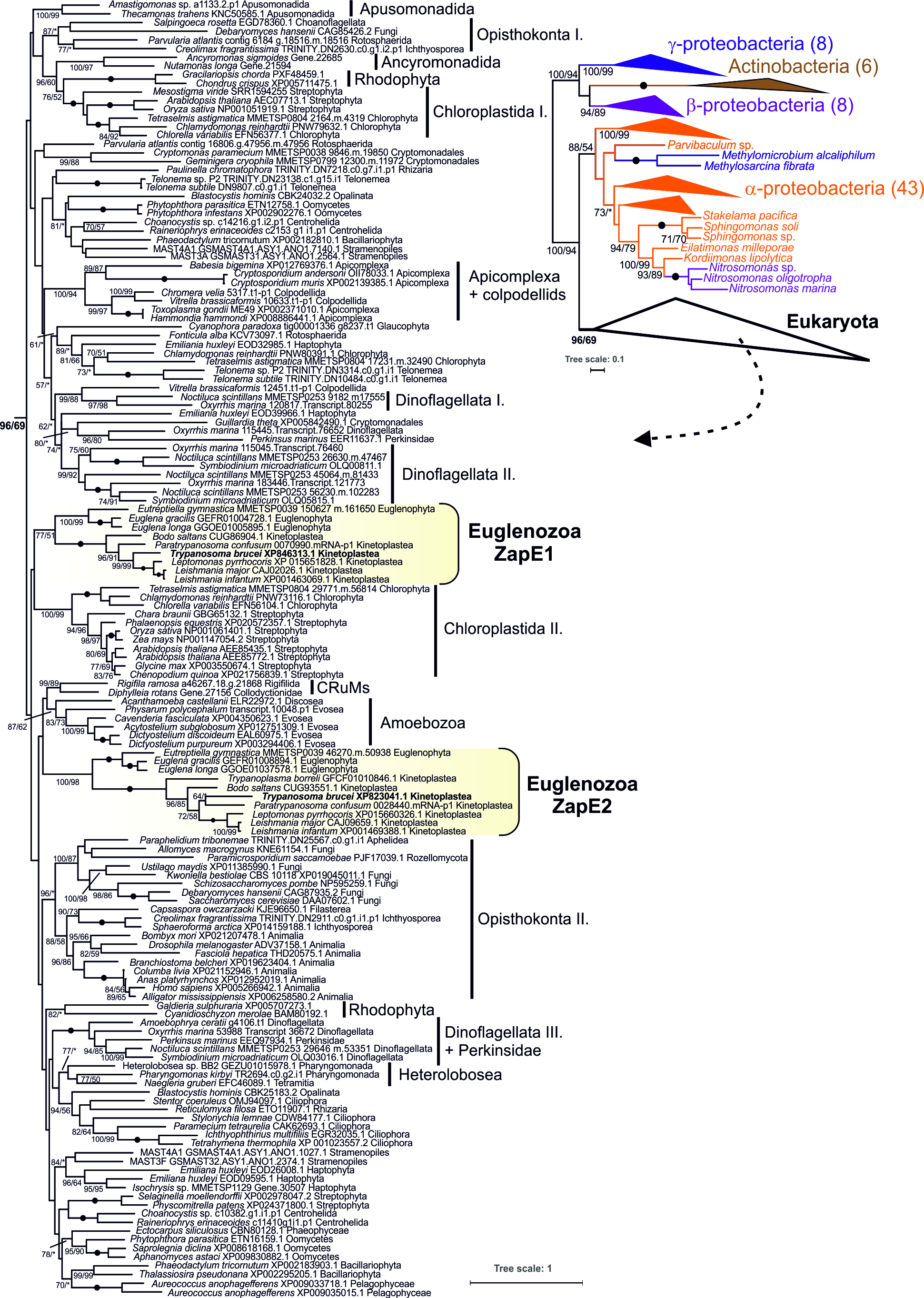

Supplement: S3 Fig — (TIF) [file pone.0234918.s003.tif]

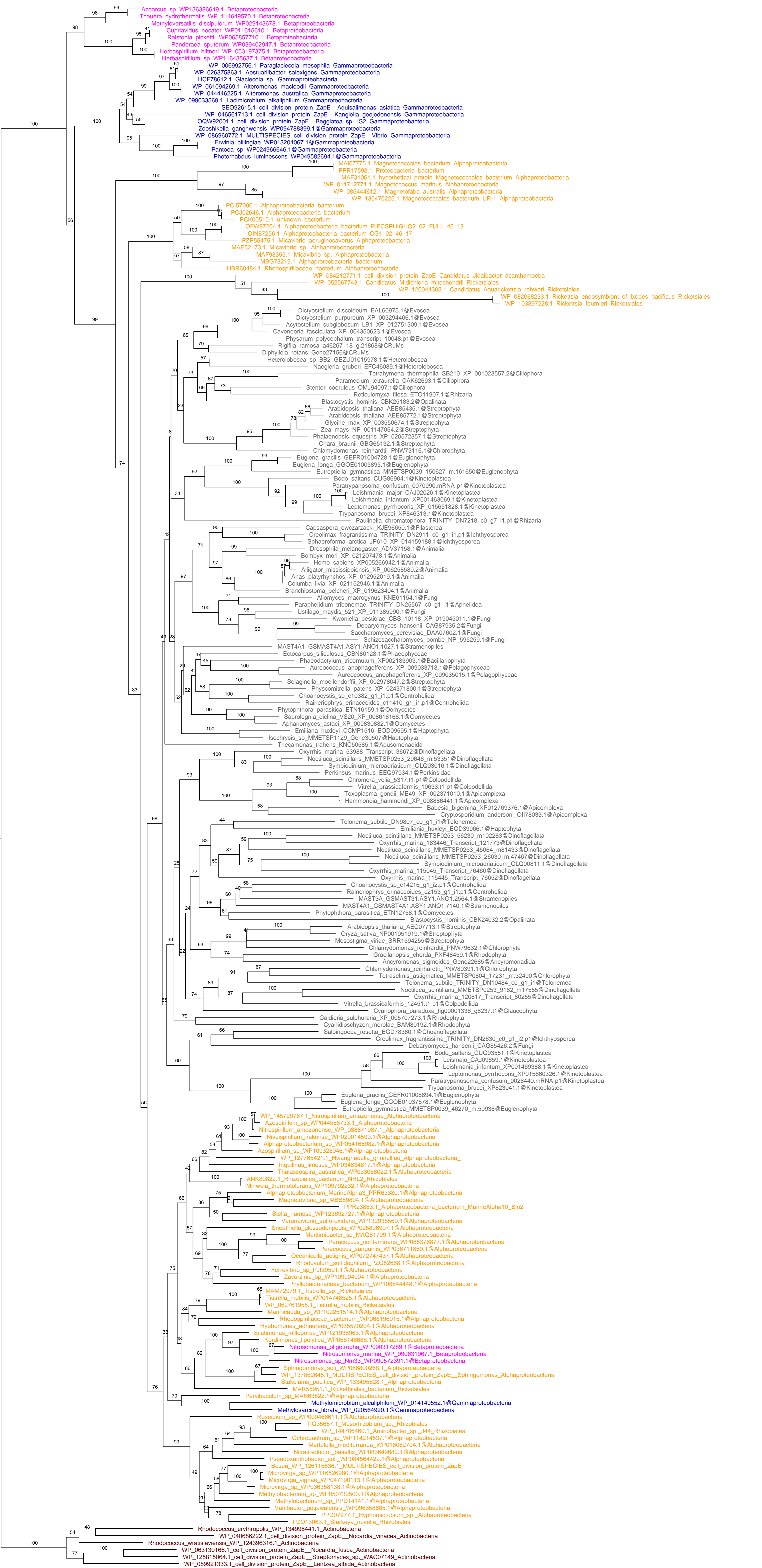

Supplement: S4 Fig — (PDF) [file pone.0234918.s004.pdf]
