# Supplementary material for: ZapE/Afg1 interacts with Oxa1 and its depletion causes a multifaceted phenotype
Source: PLoS One. 2020 Jun 24;15(6):e0234918. doi: 10.1371/journal.pone.0234918 (PMC7314023; doi:10.1371/journal.pone.0234918)

Fig 3

Double knockdown cells - western blot analysis

(samples were loaded in duplicates)

ZapE1_V5tag

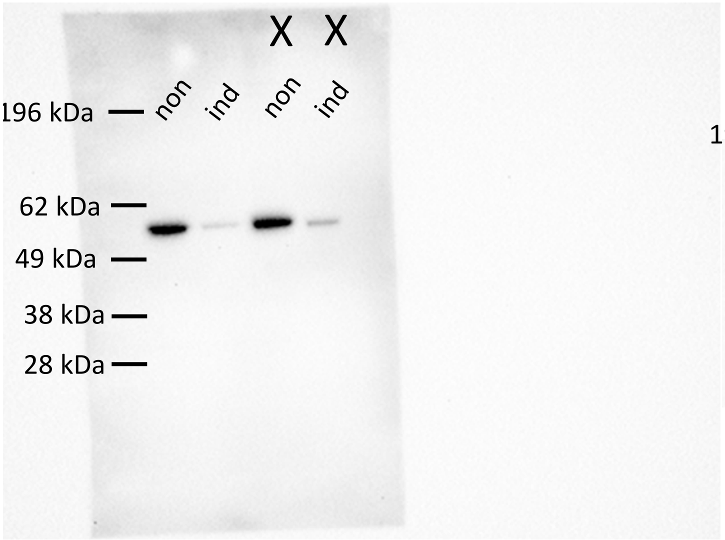

Tubulin- loading control

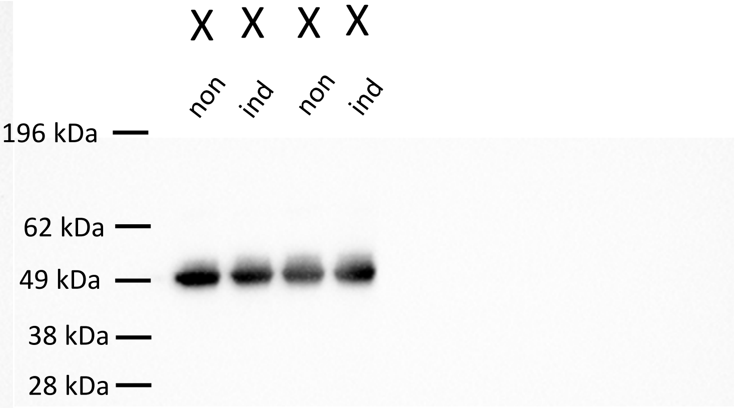

ZapE2_HAtag

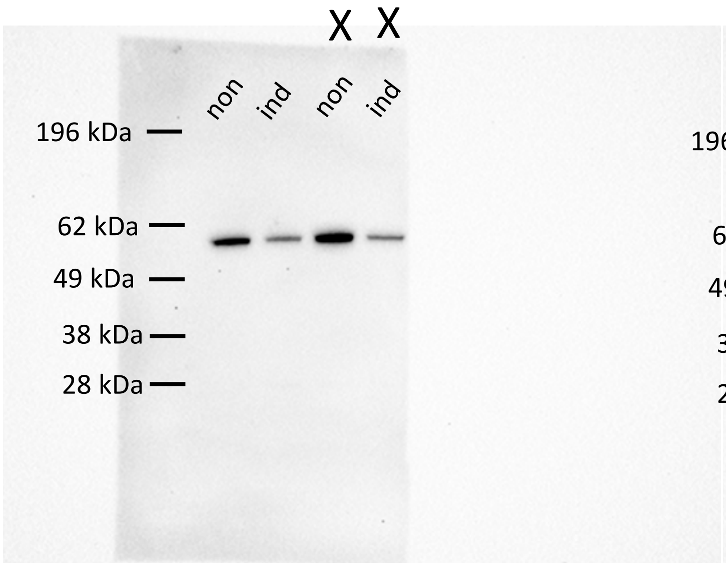

Tubulin- loading control

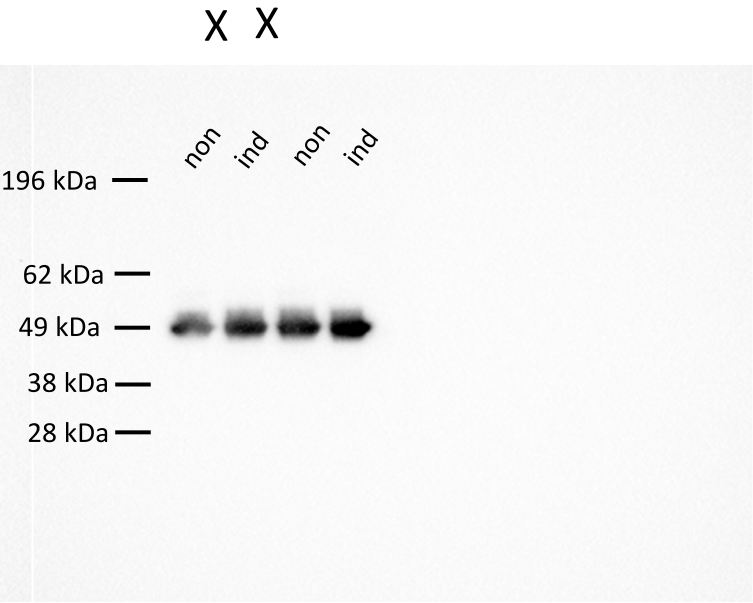

S2 Fig

ZapE1 knockdown cells western blot analysis
(samples were loaded in duplicates)

ZapE1_V5tag

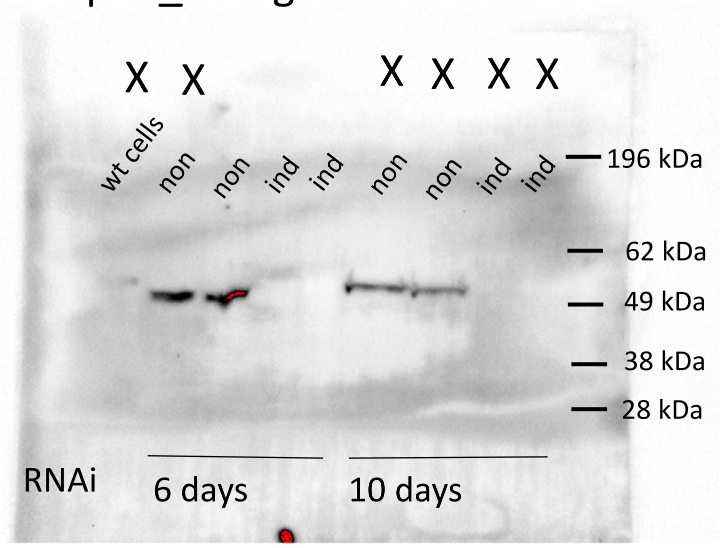

Tubulin- loading control

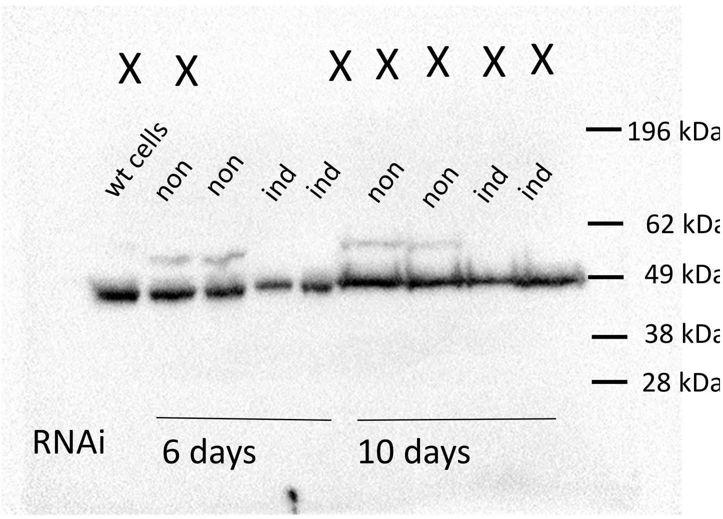

ZapE2 knockdown cells western blot analysis
(samples were loaded in duplicates)

ZapE2_V5tag

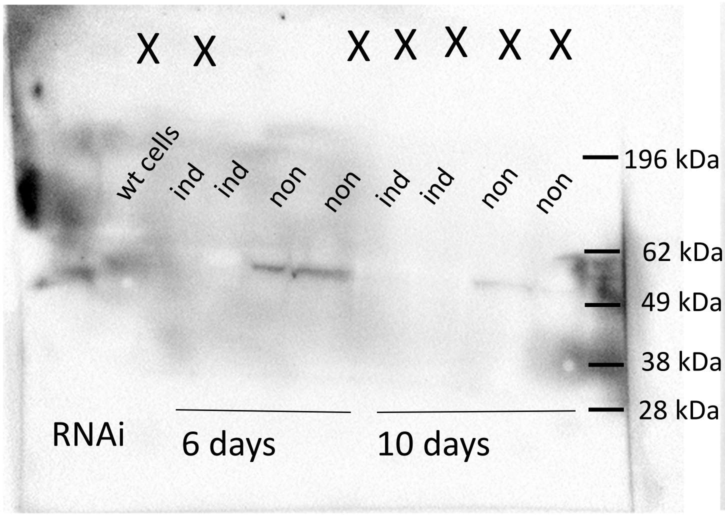

Tubulin- loading control

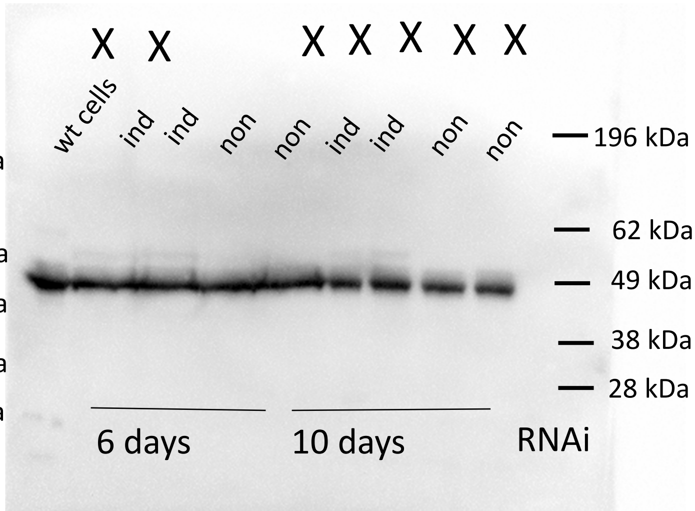

Supplement: S1 Raw Images — (PDF) [file pone.0234918.s009.pdf]
